# miR-409-3p Regulated by GATA2 Promotes Cardiac Fibrosis through Targeting Gpd1

**DOI:** 10.1155/2022/8922246

**Published:** 2022-10-12

**Authors:** Chun Wang, Shengxia Yin, Qin Wang, Min Jiang, Shanshan Li, Wen Zhen, Yi Duan, Huanyu Gu

**Affiliations:** ^1^Department of Geriatrics, Nanjing Drum Tower Hospital, The Affiliated Hospital of Nanjing University Medical School, Nanjing 210008, China; ^2^Department of Infectious Diseases, Nanjing Drum Tower Hospital, The Affiliated Hospital of Nanjing University Medical School, Nanjing 210008, China; ^3^Department of Ophthalmology, Shanghai General Hospital, Shanghai Jiao Tong University, Shanghai 200080, China

## Abstract

Cardiac fibrosis is a hallmark of numerous chronic cardiovascular diseases that leads to heart failure. However, there is no validated therapy for it. Dysregulation of microRNAs has been confirmed to be involved in cardiac fibrosis development. However, the regulatory network was not well explored. This study was the first to highlight the role and molecular mechanism of miR-409-3p in cardiac fibrosis. We found that miR-409-3p was consistently increased in three fibrotic models, including heart tissues of postmyocardial infarction (MI) mice and neonatal rat cardiac fibroblasts treated with angiotensin II (Ang II) or transforming growth factor-*β* (TGF-*β*). Furthermore, myocardial infarction surgery-induced cardiac fibrosis and dysfunction were attenuated by systemic delivery of miR-409-3p antagomir. Notably, transfection with miR-409-3p mimics promoted the proliferation of cardiac fibroblasts and fibroblast-to-myofibroblast differentiation, accompanied by upregulated expression of Col1a1, Col3a1, and *α*-SMA. On the contrary, the miR-409-3p inhibitor exhibited the opposite effect. Following this, we verified Gpd1 as a direct target of miR-409-3p. Gpd1 siRNA abolished the antifibrotic effect of miR-409-3p inhibitor in neonatal rat cardiac fibroblasts, suggesting that miR-409-3p promotes cardiac fibrosis at least partially through Gpd1. Moreover, GATA2 was identified as a cardiac fibrosis-associated upstream positive transcription factor of miR-409-3p. Finally, these findings suggest that modulating miR-409-3p could be a potential therapeutic method for cardiac fibrosis.

## 1. Introduction

Cardiac fibrosis is a pathological manifestation of myocardial remodeling, which is characterized by increased fibroblast-to-myofibroblast conversion and excessive extracellular matrix (ECM) deposition within the myocardial interstitium, leading to myocardial stiffness, ventricular dilation, arrhythmias, and heart failure [1, 2]. Myocardial fibrosis occurs in almost all chronic cardiovascular diseases [3], such as myocardial infarction (MI), which remains a leading cause of morbidity and mortality worldwide [4].

Several therapeutic agents, including angiotensin II receptor antagonist, acetylcholine esterase inhibition, and LCZ696, have been applied to partially reverse ventricular remodeling in the last few decades [5]. Nevertheless, these interventions are still far from satisfactory. Therefore, effective therapies for antifibrosis are vital to improve disease prognosis and reduce healthcare expenditure [6].

Understanding the molecular mechanisms underlying cardiac fibrosis is beneficial to the development of novel strategies for therapeutic intervention. Inflammation, oxidative stress, and aging have been proven to contribute to cardiac fibrosis, which is regulated by multiple factors [7–9]. Angiotensin II (Ang II) and transforming growth factor-*β* (TGF-*β*) are the most important among these factors. Both of them are secreted by fibroblasts or myofibroblasts. They promote the proliferation of fibroblasts, fibroblast-to-myofibroblast transdifferentiation (FMT), and ECM accumulation [10].

MicroRNA (miRNA) belongs to the family of single-stranded noncoding RNA with approximately 22 nucleotides that negatively regulates the target genes at the posttranscriptional level, which is involved in a wide range of biological processes [11]. A single mRNA is regulated by multiple miRNAs, and individual miRNA targets even hundreds of mRNAs, leading to complicated regulatory networks [12]. Accumulating studies have revealed that miRNAs that participate in the myocardial fibrotic program, such as miR-18a-5p, attenuate cardiac fibrosis via regulating the Notch2 pathway [13], and miR-24-3p alleviates myocardial fibrosis through PHB2 [14]. In addition, miR-99b-3p and miR-223 were reported to act as fibrogenic factors [15, 16]. These observations demonstrated that miRNAs might be promising therapeutic targets for alleviating cardiac fibrosis. miR-409-3p, an unexplored candidate, has been primarily investigated in oncology. For instance, miR-409-3p promotes epithelial to mesenchymal transition and tumor growth in prostate cancer and also accelerates osteoblastic differentiation through the Wnt/*β*-catenin pathway [17, 18], a crucial pathway in regulating cardiac fibrosis. Previous clinical study discovered its potential value in distinguishing atrial fibrillation [19]. Moreover, the circulating miR-409-3p was significantly downregulated in mitral regurgitation patients with heart failure [20]. Nonetheless, there is little evidence about the role of miR-409-3p in cardiac fibrosis currently.

Glycerol-3-phosphate dehydrogenase 1 (GPD1) regulates glycerol metabolism by transporting equivalents across the mitochondrion membrane [21]. It was suggested to cause an antitumor effect in breast cancer [22] and inhibit the progression of renal clear cell carcinoma through lipid metabolism [23]. A clinical research also demonstrated that mutant Gpd1 caused infantile hepatic fibrosis [24]. GATA-binding protein 2 (GATA2) is a zinc finger transcription factor that belongs to the GATA family. GATA2 is mainly involved in the proliferation and development of hematopoietic and endocrine cell lineages [25] and promotes the proliferation of vascular smooth muscle cells [26]. GATA2 deficiency was found be involved in pulmonary fibrosis [27]. To our knowledge, little is known about whether Gpd1 or GATA2 participated in cardiac fibrosis.

This study investigated the effect of miR-409-3p on cardiac fibrosis and the underlying mechanism in vivo and in vitro. We found that miR-409-3p levels were significantly high in the infarct zone of the heart in MI modeling mice. Our data demonstrated that treatment with miR-409-3p antagomir could attenuate myocardial fibrosis and improve cardiac function in post-MI mice. Furthermore, at the cellular level, overexpression of miR-409-3p increased the fibrotic activity of cardiac fibroblasts, whereas miR-409-3p knockdown had the opposite effect. We further identified GPD1 as a direct target of miR-409-3p and GATA2 as an upstream regulatory gene of miR-409-3p. Collectively, these findings are the first to demonstrate that miR-409-3p promotes cardiac fibrosis by interfering with Gpd1. Meanwhile, miR-409-3p is positively regulated by GATA2 in the process of cardiac fibrosis, thus providing a potential therapeutic target for treating cardiac fibrosis.

## 2. Materials and Methods

### 2.1. Ethics Statement

Animals were maintained in the Laboratory Animal Center of Nanjing Drum Tower Hospital (Nanjing, China). All procedures followed the Guide for the Care and Use of Laboratory Animals published by the US National Institutes of Health (8th Edition, National Research Council, 2011). The experimental protocols were approved by the ethical committee of Nanjing Drum Tower Hospital (Approval No. 2021AE01062).

### 2.2. Animal Models

C57BL/6 mice (male; 8 weeks; 20-25 g) were subjected to sham or MI surgery. After anesthetizing the mice, the hearts were exposed, and the left anterior descending artery was ligated with a 7-0 silk suture, while sham-operated mice underwent the same procedure but without ligation. Mice were all sacrificed three weeks post-MI. Before the surgery, mice were injected with 80 mg/kg miR-409-3p antagomir or negative control (nc) antagomir (RiboBio, Guangzhou, China) via tail vein for three consecutive days.

### 2.3. Sequencing Analysis

Total RNA was isolated from 50 mg ventricular tissues of sham or MI mice to construct a complementary DNA library (Xu Ran Biotechnology Co., Shanghai, China). VAHTS Small RNA Library Prep Kit for Illumina (Vazyme, Nanjing, China) was used to prepare miRNA libraries. VAHTS Universal V6 RNA-seq Library Prep Kit for Illumina (Vazyme) was used to prepare mRNA libraries. The resulting libraries were concentrated with ethanol precipitation and quantified by the Qubit 2.0 Fluorometer (Thermo Fisher, Waltham, MA, USA) before sequencing on the NovaSeq 6000 sequencer (Illumina, San Diego, CA, USA). Differentially expressed miRNAs or mRNAs were identified with DESeq software 1.34.1. Gene expressions of more than 2-fold change compared with the sham group were considered significantly upregulated or downregulated.

### 2.4. Echocardiography

Three weeks after the surgery, mice were anesthetized with isoflurane (1.5-2%), and then, M-mode echocardiography was taken by an ultrasound Vevo 3100 system (VisualSonics Inc., Toronto, Ontario, Canada) equipped with a 30 MHz high-frequency scan head. Ejection fraction (EF), fractional shortening (FS), diastolic left ventricle internal diameter (LVID;d), and systolic left ventricle internal diameter (LVID;s) were measured at the papillary muscle levels along the long cardiac axis.

### 2.5. Masson's Trichrome Staining

Myocardium specimens from sham or post-MI mice were fixed in 4% paraformaldehyde for 24 h, embedded in paraffin, and then sectioned at 5 *μ*m. The sections were dewaxed with xylene (5 min × 3) and infused in an alcohol gradient. After that, these sections were stained with the Masson kit (Keygen, Nanjing, China). Images were taken using an optical microscope (Olympus BX43, Japan). The fibrotic level was quantified by calculating the percentage of the blue area (collagen staining)/total left ventricular area with ImageJ analysis software (National Institutes of Health, Bethesda, MD, USA).

### 2.6. Isolation, Culture, Transfection, and Chemical Treatment of NRCF

Neonatal rat cardiac fibroblasts (NRCFs) were isolated from newborn SD rats (1-3 day). First, minced ventricle tissues were digested in the buffer of 40% collagenase and 60% trypsin (BioFroxx, Guangzhou, China). Then, NRCFs were separated from cardiomyocytes by gravity separation for 2 h, attached to the 10 cm cell culture dishes, and cultured with medium (DMEM+10% fetal bovine serum+1% streptomycin+1% penicillin) (Gibco, Grand Island, CA, USA). Cells were kept at 37°C in humid air containing 5% CO_2_.

Cells at the second passage were exposed to miR-409-3p mimic (100 nM)/inhibitor (200 nM) versus the corresponding nc mimic/nc inhibitor (RiboBio) for 48 hours in culture medium with 1% fetal bovine serum (FBS). At the same time, cells were treated with Ang II (200 nM, 48 h, Sigma, St. Louis, MO, USA) or recombinant human TGF-*β*1 (20 ng/mL, 48 h, PeproTech, Rocky Hill, NJ, USA). Reagents above including Gpd1 siRNA, GATA2 siRNA, and nc siRNA (50 nM, 48 h, Sangon, Shanghai, China) were all transfected with Lipofectamine 3000 (Invitrogen, Carlsbad, CA, USA) according to the manufacturer's instructions.

### 2.7. RNA Extraction and Quantitative Real-Time Polymerase Chain Reaction (qRT-PCR)

Total RNAs were extracted from heart tissues or cells using TRIzol reagent (Invitrogen) according to the manufacturer's instructions. 400 ng RNAs were reversely transcribed with PrimeScript RT reagent kit (Takara, Tokyo, Japan) to obtain cDNAs. QuantStudio Real-Time PCR Instrument (Thermo Fisher) with Bio-Rad SYBR qPCR (Bio-Rad, Hercules, CA, USA) was used to detect mRNA expression. The primers are listed in Supplementary Table 1. 18S was used as an endogenous control. Bulge-Loop™ miRNA qPCR Primer Set (RiboBio) was used to determine miRNA expression levels. 5S was used for miRNA template normalization.

### 2.8. Western Blot Analysis

Heart tissues or NRCFs were lysed in RIPA buffer containing protease inhibitor cocktail (Keygen). Bicinchoninic Acid Protein Assay Kit (Thermo Fisher) was used to evaluate protein concentration. First, equal amounts of proteins were separated by sodium dodecyl sulfate-polyacrylamide gel electrophoresis (SDS-PAGE) and transferred onto polyvinylidene fluoride (PVDF) membranes (Millipore, Billerica, MA, USA). Subsequently, the membranes were incubated with primary antibodies overnight at 4°C, followed by incubating with peroxidase- (HRP-) conjugated secondary antibody for 2 hours at room temperature. Finally, the protein signal was visualized with an ECL kit (Tanon, Shanghai, China) and detected by GelCap Software (Tanon-5200 Multi). All antibodies are listed in Supplementary Table 2.

### 2.9. Immunofluorescent Staining

NRCFs were fixed in 4% paraformaldehyde (20 min) and permeabilized with 0.2% Triton X-100 (10 min), followed by blocking (2 h) with 5% bovine serum albumin (BSA) and incubating with the *α*-SMA-Cy3 antibody (1 : 200, 2 h, Sigma) at room temperature. EdU assay was performed with a kFluor488 Click-iT EdU kit (Keygen). Cell nuclei were labeled with DAPI (1 *μ*g/mL, 15 min, Keygen). Calculating EdU-positive cell number/DAPI-positive cell number indicated the NRCF proliferation. Fluorescence intensity of *α*-SMA was calculated to indicate the differentiation ability of NRCF.

5 *μ*m slices of heart tissues were cut, the remaining steps were the same as above, and antibodies used for slices include *α*-SMA-Cy3 antibody, Vimentin antibody (1 : 100, 12 h, Abclonal, Wuhan, China), and Ki67 antibody (1 : 100, 12 h, Abclonal). Eight fields/sample (×100 magnification) were viewed under the fluorescence microscope (Leica Microsystem Thunder Imager, Germany). Calculating Ki67/Vimentin double-positive cell numbers indicated the cardiac fibroblast proliferation. Calculating *α*-SMA/Vimentin double-positive cell numbers indicated the cardiac fibroblast differentiation.

### 2.10. Luciferase Reporter Gene Assay

To get wild-type or mutant GPD1-luc vectors, 3′UTR nucleotides of GPD1 containing wild-type or mutated miR-409-3p binding sites were synthesized and cloned into pmirGLO Vector (Promega, Madison, WI, USA). 293T cells were harvested after transfection for 48 h. Dual-Luciferase Reporter Assay System (Promega) was used to measure luciferase activity according to the manufacturer's instructions.

### 2.11. MicroRNA Target and Upstream Gene Prediction

Three miRNA target prediction websites including miRDB (http://mirdb.org/), TargetScan (https://www.targetscan.org/), and miRWalk (http://129.206.7.150/) were used for predicting potential targets of miR-409-3p. We focused on the intersection of the three databases, picking the most likely target gene based on the mRNA sequencing results. Besides, TransmiR v2.0 database (http://www.cuilab.cn/transmir) was used to search for the upstream regulatory factor of miR-409-3p.

### 2.12. Statistical Analysis

In each experiment, data were displayed as mean ± standard deviation (SD) of at least three independent experiments. The Mann–Whitney test was used to analyze the differences between two groups. The Kruskal-Wallis test was chosen for multiple comparisons. Data were considered statistically significant when the *P* value was less than 0.05. Statistical analysis was performed by SPSS 20.0 (IBM, USA) and GraphPad Prism 6 software (GraphPad Software Inc., USA).

## 3. Results

### 3.1. miR-409-3p Expression Is Upregulated in Cardiac Fibrosis

**Figure 1:**
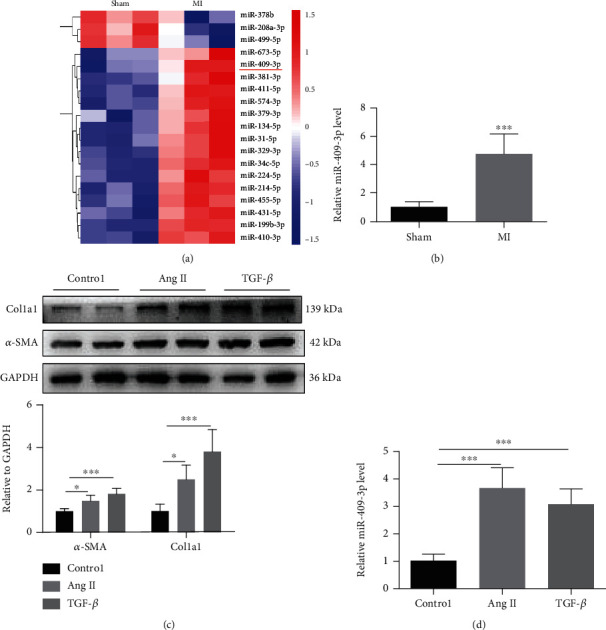
miR-409-3p expression is upregulated in cardiac fibrosis. (a) Dysregulated miRNAs in heart tissues of post-MI mice versus sham mice whose roles in cardiac fibrosis are unknown (*n* = 3). (b) miR-409-3p expression of post-MI mice versus sham mice determined by qRT-PCR (*n* = 5). (c, d) Protein levels of fibrotic biomarkers and miR-409-3p expression in two cell models of cardiac fibrosis induced by Ang II or TGF-*β* (*n* = 5). ^∗^*P* < 0.05 and ^∗∗∗^*P* < 0.001 versus respective controls.

To evaluate which miRNAs might be involved in cardiac fibrosis, miRNA sequencing was performed to determine miRNA expression significance between sham and post-MI ventricles. Compared to the sham group, over 100 miRNAs were dysregulated more than 2-folds (*P* < 0.05) in the MI group. In total, 104 miRNAs were upregulated, and 34 were downregulated. Some miRNAs have been associated with cardiac fibrosis, such as miR-214-3p and miR-210-5p [28, 29]. In addition, we chose 19 other miRNAs (Figure 1(a) and Supplementary Table 3) that have not been previously reported participating in myocardial fibrosis according to our assay. Among these miRNAs, miR-409-3p was increased by more than four folders, which was considered related to tumorigenesis in tumor-associated studies. In this study, we further explored its role in cardiac fibrosis.

We reconfirmed that miR-409-3p was significantly upregulated in heart tissues of MI mice by qRT-PCR analysis (Figure 1(b)). Furthermore, we applied two different cell models; one was Ang-II-induced cardiac fibrosis on cultured NRCFs, and another one was TGF-*β* induced for further study. In both models, the protein expressions of the two fibrotic markers, type I collagen alpha 1 (Col1a1) and alpha-smooth muscle actin (*α*-SMA), significantly increased (Figure 1(c)). Additionally, miR-409-3p was also upregulated in the two cell models (Figure 1(d)). Taken together, these data suggest a potential role of miR-409-3p in cardiac fibrosis.

### 3.2. Deficiency of miR-409-3p Reverses MI-Induced Cardiac Fibrosis and Cardiac Dysfunction

**Figure 2:**
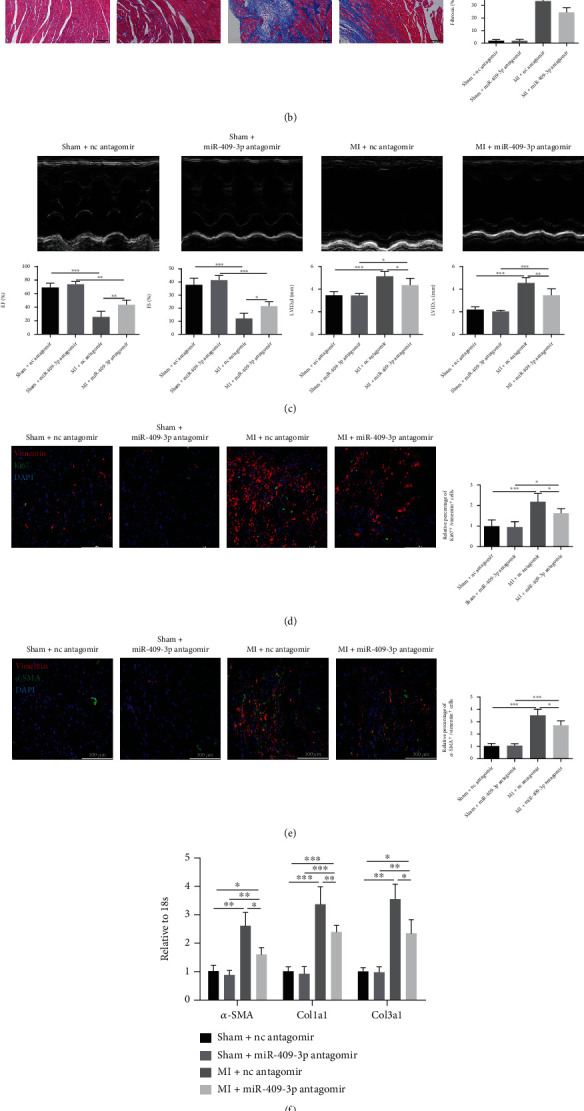
Deficiency of miR-409-3p reverses MI-induced cardiac fibrosis and cardiac dysfunction. C57BL/6 mice were injected with miR-409-3p antagomir or nc antagomir via tail vein for three consecutive days and subjected to sham or MI surgery for 3 weeks. (a) The expression of miR-409-3p in heart tissues measured by qRT-PCR. (b) Masson's trichrome staining and quantification of collagen deposition in heart sections. (c) Cardiac function recorded by echocardiography. (d) Proliferation of cardiac fibroblasts determined by immunofluorescent staining of Ki67 and Vimentin. (e) Fibroblast-to-myofibroblast differentiation determined by immunofluorescent staining of *α*-SMA and Vimentin. (f) Expression of fibrotic markers (*α*-SMA, Col1a1, and Col3a1) measured by qRT-PCR. (g) Protein levels of CTGF, *α*-SMA, Col1a1, and Col3a1 in heart tissues (*n* = 5). Scale bar: 100 *μ*m. ^∗^*P* < 0.05, ^∗∗^*P* < 0.01, and ^∗∗∗^*P* < 0.001 versus respective controls.

We hypothesized that miR-409-3p was implicated in the process of cardiac fibrosis. Therefore, we investigated its role in vivo by administrating nc antagomir or miR-409-3p antagomir into C57BL/6 mice via tail vein injection for three consecutive days and subjecting them to sham or MI surgery. The mice were sacrificed three weeks after the surgery, and the miR-409-3p expression in heart tissues was assessed by qRT-PCR. The results demonstrated that miR-409-3p was efficiently downregulated by antagomir (Figure 2(a)). As Masson's staining images are displayed in Figure 2(b), the myocardia were disordered, and the structures of ventricles were severely disrupted in post-MI hearts than the sham group. More importantly, collagen depositions were evident in the MI group, while inhibition of miR-409-3p attenuated these phenomena (Figure 2(b)). In addition, we found that inhibition of miR-409-3p improved MI-induced cardiac dysfunction, including decreased EF and FS, and increased LVID assessed by echocardiography (Figure 2(c)).

Since the proliferation of fibroblasts and FMT are two important events in the progression of cardiac fibrosis, we used cardiac sections' immunofluorescence staining to search for the effects of miR-409-3p antagomir. Vimentin was used to label fibroblasts, proliferative cells were labeled by staining the Ki67 antibody, and FMT was indicated by *α*-SMA staining. Inhibition of miR-409-3p attenuated fibroblast proliferation and FMT since the numbers of Ki67/Vimentin or *α*-SMA/Vimentin double-positive cells were decreased after treatment with miR-409-3p antagomir (Figures 2(d) and 2(e)). Moreover, miR-409-3p inhibition lowered MI-induced upregulation of fibrosis-related genes including *α*-SMA, Col1a1, and Col3a1 (Figure 2(f)). Meanwhile, protein levels of connective tissue growth factor (CTGF), *α*-SMA, Col1a1, and Col3a1 revealed a similar variation tendency (Figure 2(g)). These in vivo results implied that deficiency of miR-409-3p attenuates cardiac fibrosis in mice under MI surgery.

### 3.3. miR-409-3p Promotes Cardiac Fibrosis In Vitro

**Figure 3:**
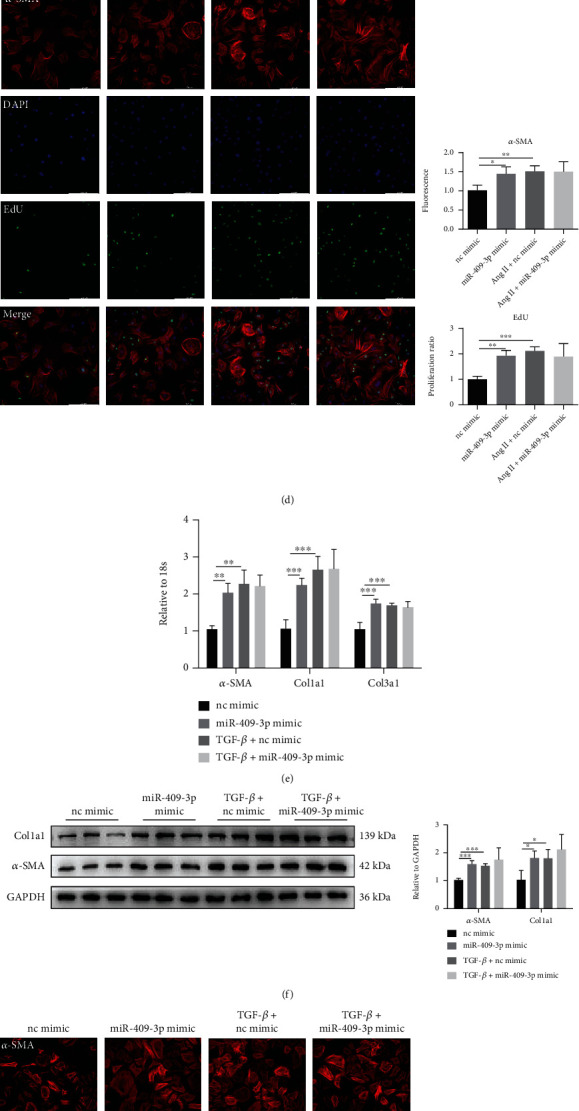
miR-409-3p mimic promotes cardiac fibrosis in vitro. (a) Expression of miR-409-3p in cultured NRCFs transfected with miR-409-3p mimic (*n* = 5). (b, c) mRNA and protein levels of fibrosis-related genes in NRCFs transfected with miR-409-3p mimic and subjected to Ang II stimulation (*n* = 5 for qRT-PCR, *n* = 6 for western blot). (d) Proliferation and differentiation degree of NRCFs determined by immunofluorescent staining of EdU and *α*-SMA (*n* = 5). (e, f) Expression of fibrotic genes in NRCFs transfected with miR-409-3p mimic in the presence or absence of TGF-*β* treatment (*n* = 5 for qRT-PCR, *n* = 6 for western blot). (g) Proliferation and differentiation degree of NRCFs evidenced with EdU/*α*-SMA staining (*n* = 5). Scale bar: 200 *μ*m. ^∗^*P* < 0.05, ^∗∗^*P* < 0.01, and ^∗∗∗^*P* < 0.001 versus respective controls.

We next sought to investigate the effect of miR-409-3p on cardiac fibrosis in vitro with NRCFs. The miR-409-3p level was significantly increased by transfecting the miR-409-3p mimic (Figure 3(a)). In addition, forced expression of miR-409-3p could enhance mRNA levels of *α*-SMA, Col1a1, and Col3a1. However, overexpression of miR-409-3p failed to further increase the levels of these genes in cell models stimulated by Ang II (Figure 3(b)). Consistent with this, western blot results revealed that protein levels of *α*-SMA and Col1a1 were upregulated by miR-409-3p mimic, however not in the case of Ang II treatment (Figure 3(c)). Moreover, we employed EdU (5-ethynyl-2′-deoxyuridine) staining to indicate cell proliferation and *α*-SMA fluorescence intensity to determine the degree of FMT. As expected, miR-409-3p mimic promoted the proliferation and differentiation ability of NRCF without Ang II treatment (Figure 3(d)). To further confirm the association, we reached the same conclusions as above in another cardiac fibrosis cell model in the presence of TGF-*β* stimulation (Figures 3(e)–3(g)).

**Figure 4:**
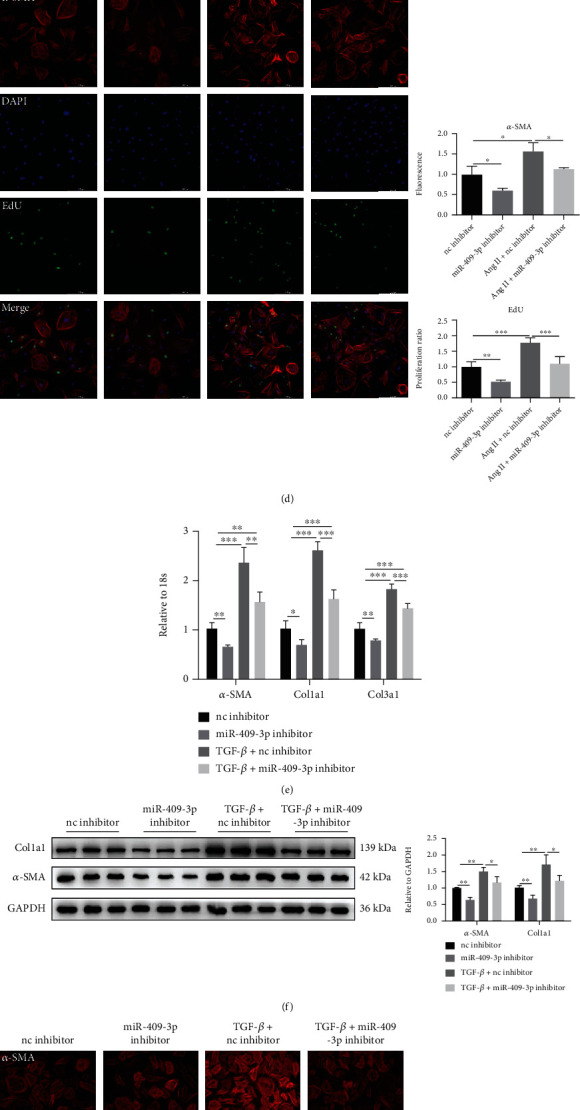
Inhibition of miR-409-3p suppresses cardiac fibrosis in NRCF. (a) miR-409-3p level in NRCFs transfected with miR-409-3p inhibitor measured by qRT-PCR (*n* = 5). (b, c) qRT-PCR and western blot were, respectively, conducted to detect levels of fibrosis-related genes in NRCFs transfected with miR-409-3p inhibitor and subjected to Ang II stimulation (*n* = 5 for qRT-PCR, *n* = 6 for western blot). (d) Proliferation and differentiation degree of NRCFs determined by immunofluorescent staining of EdU and *α*-SMA (*n* = 5). (e, f) Expression of fibrotic genes in NRCFs transfected with miR-409-3p inhibition in the presence or absence of TGF-*β* treatment (*n* = 5 for qRT-PCR, *n* = 6 for western blot). (g) NRCF proliferation and differentiation into myofibroblasts evidenced by EdU/*α*-SMA staining (*n* = 5). Scale bar: 200 *μ*m. ^∗^*P* < 0.05, ^∗∗^*P* < 0.01, and ^∗∗∗^*P* < 0.001 versus respective controls.

Besides the effects of miR-409-3p mimic on cardiac fibrosis, the miR-409-3p inhibitor was also studied. qRT-PCR results demonstrated that the inhibitor effectively reduced the content of miR-409-3p (Figure 4(a)). Furthermore, contrary to the effects of mimic, miR-409-3p inhibitor markedly downregulated the mRNA and protein expression of fibrosis-related genes both at the basal condition and after Ang II or TGF-*β* stimulation (Figures 4(b), 4(c), 4(e), and 4(f)). Moreover, inhibition of miR-409-3p also decreased the proliferation and differentiation capacity of NRCF (Figures 4(d) and 4(g)). The observations in vitro are consistent with the previous study in vivo, claiming that miR-409-3p can promote cardiac fibrosis.

### 3.4. miR-409-3p Affects Cardiac Fibrosis by Targeting Gpd1

**Figure 5:**
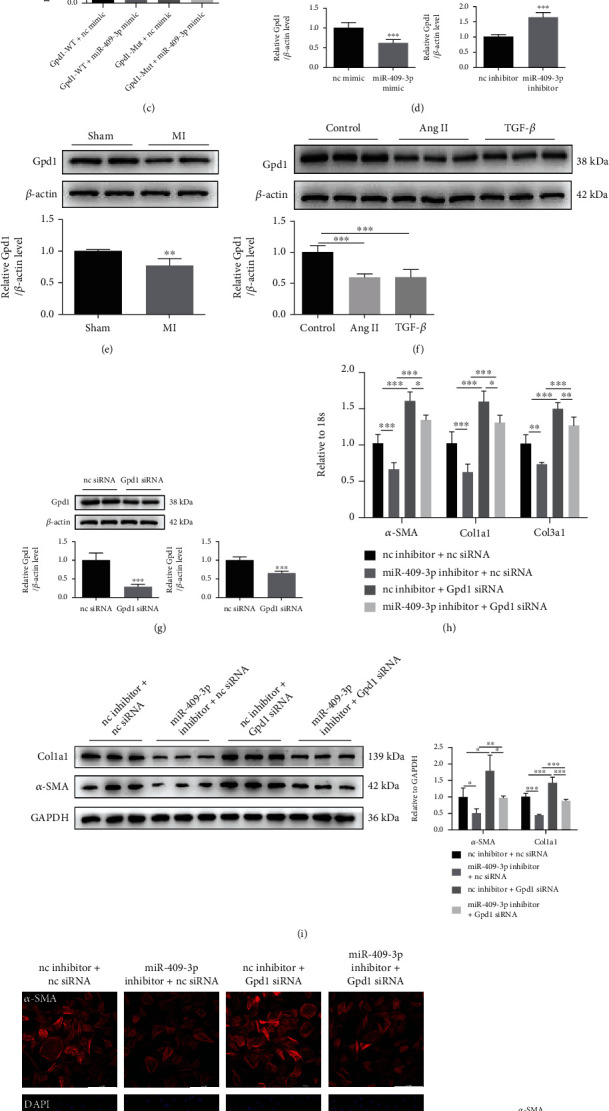
miR-409-3p affects cardiac fibrosis by targeting Gpd1. (a) Three prediction databases were used to search for the potential target gene of miR-409-3p. (b) Dysregulated genes in heart tissues of post-MI mice versus sham mice (*n* = 3). (c) Direct target of miR-409-3p identified by luciferase reporter assays (*n* = 3). (d) Protein levels of Gpd1 in NRCFs treated with miR-409-3p mimic or inhibitor (*n* = 6). (e) Expression of Gpd1 in heart tissues from post-MI mice versus sham mice detected by western blot (*n* = 5). (f) Protein levels of Gpd1 in two cell models of cardiac fibrosis induced by Ang II or TGF-*β* (*n* = 6). (g) Silence efficiency of Gpd1 siRNA detected by qRT-PCR and western blot (*n* = 5). (h–j) The effect of Gpd1 knockdown in NRCFs transfected with miR-409-3p inhibitor on fibrotic gene expression, cell proliferation, and differentiation into myofibroblasts (*n* = 5 for qRT-PCR and immunofluorescent staining, *n* = 6 for western blot). Scale bar: 200 *μ*m. ^∗^*P* < 0.05, ^∗∗^*P* < 0.01, and ^∗∗∗^*P* < 0.001 versus respective controls.

To look into the molecular mechanism of miR-409-3p in the process of fibrosis, we selected the intersection of predicted results from three databases to search for the potential target gene of miR-409-3p (Figure 5(a)). Among the 36 candidates (Supplementary Table 4), Gpd1 was downregulated in the mRNA sequencing analysis (Figure 5(b) and Supplementary Table 5), which was performed to determine mRNA expression significance between sham and post-MI ventricles. Next, we verified whether Gpd1 is a fibrosis-associated direct target gene of miR-409-3p. The bioinformatic analysis presented that Gpd1 had a miR-409-3p binding site in its 3′UTR region (Figure 5(c)). Luciferase reporter assay demonstrated that miR-409-3p inhibited the luciferase activity of wild-type 3′UTR construct for Gpd1, whereas it had no impact on the luciferase activity of the mutant binding site (Figure 5(c)), indicating that Gpd1 is a direct target gene of miR-409-3p.

Since miRNAs work through negative regulation of target mRNAs, we next found that the protein level of Gpd1 was suppressed by miR-409-3p mimic and enhanced by miR-409-3p inhibitor in NRCF (Figure 5(d)), further confirming that miR-409-3p can negatively regulate Gpd1. Contrary to the results of miR-409-3p in Figure 1, the protein expression of Gpd1 was decreased in the heart tissues of post-MI mice than sham mice (Figure 5(e)). The same trends occurred in fibrosis models in vitro induced by Ang II or TGF-*β* (Figure 5(f)).

Before further clarifying the role of Gpd1 in cardiac fibrosis, we proved that the designed Gpd1 siRNA effectively silenced the content of Gpd1 both at mRNA and protein levels (Figure 5(g)). Furthermore, the following functional reversal research illustrated that knockdown of Gpd1 via siRNA abolished the antifibrotic effect of the miR-409-3p inhibitor on NRCF (Figures 5(h)–5(j)). Based on these results, we conclude that Gpd1 is a novel cardiac fibrosis-associated target gene of miR-409-3p.

### 3.5. GATA2 Is Identified as a Regulatory Factor of miR-409-3p

**Figure 6:**
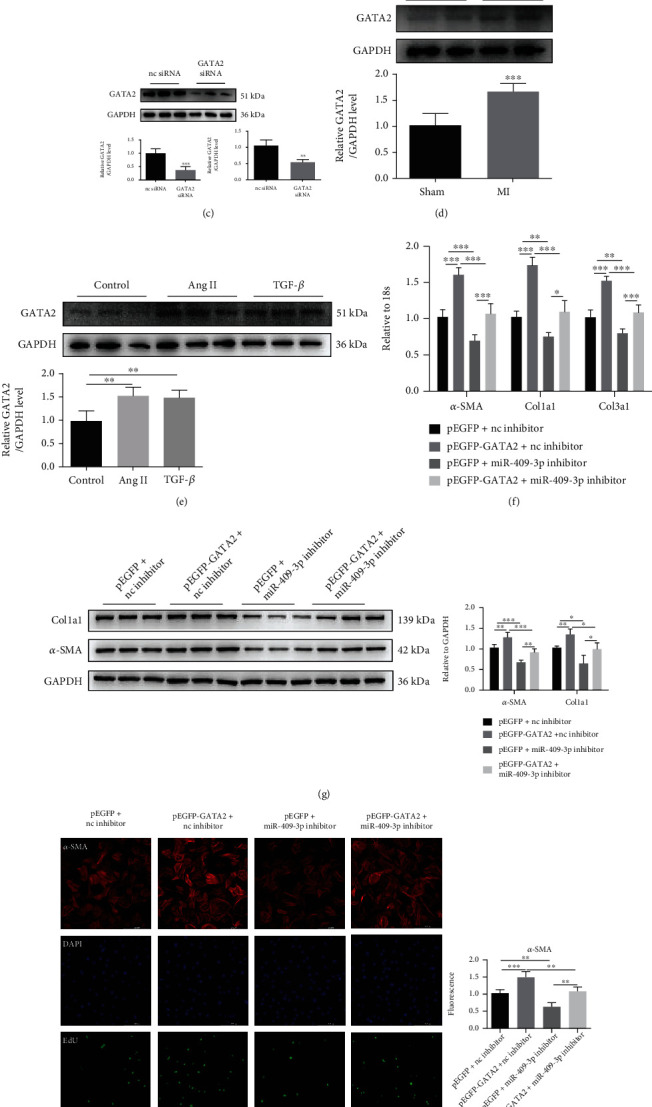
GATA2 is a fibrosis-associated positive regulatory transcription factor of miR-409-3p. (a) Transcription factors of miR-409-3p were searched in the TransmiR database. (b, c) miR-409-3p expression in NRCFs transfected with GATA2 overexpression plasmid or siRNA (*n* = 5). (d) Expression of GATA2 in heart tissues from post-MI mice compared to sham mice (*n* = 5). (e) Protein levels of GATA2 in NRCFs treated with Ang II or TGF-*β* (*n* = 6). (f–h) The effect of Gpd1 knockdown in NRCFs overexpressing GATA2 on fibrotic gene expression, cell proliferation, and differentiation into myofibroblasts (*n* = 5 for qRT-PCR and immunofluorescent staining, *n* = 6 for western blot). Scale bar: 200 *μ*m. ^∗^*P* < 0.05, ^∗∗^*P* < 0.01, and ^∗∗∗^*P* < 0.001 versus respective controls.

Transcription factor (TF) is a kind of regulatory protein involved in the regulation of transcription initiation. Relationships between TF-mRNA and miRNA-mRNA were widely studied. However, miRNA can also be dominated by TF [30]. The underlying mechanisms of many diseases were elusive, partly due to insufficient knowledge of TF-miRNA regulation. In this study, we continued exploring the potential TF of miR-409-3p by TransmiR database, a database of regulatory networks between TFs and miRNAs sorted out from literature. GATA2 is a positive regulatory TF found in cardiovascular tissues and is one of the candidate upstream TFs (Figure 6(a)). Thus, we would focus on whether GATA2 is a cardiac fibrotic TF by moderating miR-409-3p in NRCF.

GATA2 plasmid and siRNA were used to determine the regulatory effect of GATA2 on miR-409-3p. Results illustrated that GATA2 overexpression increased the miR-409-3p level. Meanwhile, downregulation of GATA2 had the opposite effect (Figures 6(b) and 6(c)). In parallel to the trends of miR-409-3p, protein levels of GATA2 were raised in heart samples of mice undergoing MI surgery and in NRCFs treated with Ang II or TGF-*β* (Figures 6(d) and 6(e)). Additionally, inhibition of the miR-409-3p attenuated the profibrotic effect of GATA2 overexpression plasmid on NRCF (Figures 6(f)–6(h)). These data provided evidence that GATA2 is a fibrosis-associated positive regulatory TF of miR-409-3p.

## 4. Discussion

Cardiac fibrosis is a crucial pathological change of ventricular remodeling and a common consequence of multitudinous cardiovascular diseases, resulting in stiffer tissue and cardiac dysfunction. Therefore, effective antifibrotic therapies are essential to improving prognoses of these diseases [31]. Unfortunately, the current medicines have not been sufficiently successful in treating cardiac fibrosis. In the past decade, numerous miRNAs have been closely linked with various pathophysiological processes. Thus, disease-associated miRNAs as therapeutic targets are emerging, including antifibrotic therapies [32].

Previous studies on miR-409-3p mainly focused on the field of the tumor, such as contributing to tumor growth in prostate cancer [18], while inhibiting proliferation and migration of osteosarcoma as a tumor suppressor [33]. These contradictory conclusions indicated that miR-409-3p plays complex tissue-based specific roles in various tumor cells. However, little was known about its effect on cardiac fibrosis. Our research was the first to highlight the role of miR-409-3p in cardiac fibrosis, both in vivo and in vitro. In the present study, miR-409-3p was found to increase in the myocardium of post-MI mice and NRCFs treated with Ang II or TGF-*β*, and this reminded us that upregulation of miR-409-3p might be a feature of cardiac fibrosis.

We discovered in vivo that miR-409-3p inhibition attenuated MI-induced cardiac dysfunction and myocardial fibrosis, accompanied by decreased ECM expression. miR-409-3p antagomir was administrated via tail vein to knock down systemic miR-409-3p. Although we have proved that miR-409-3p and cardiac fibrosis are directly related, more powerful gain- and lose-of-functional experiments are needed to confirm further this relationship, such as using transgenic mice with cardiac fibroblast-specific overexpression of miR-409-3p and cardiac fibroblast-specific miR-409-3p conditional knockout mice to study the effect of miR-409-3p on myocardial fibrosis specifically. The heart tissues we extracted were 3 weeks after MI surgery. However, fibrosis is a chronic and complicated process. Therefore, studies at various time points (early, intermediate, and late stages) to determine whether the role of miR-409-3p is time-dependent are required because determining the time point of miR-409-3p-related treatment in the future is critical. Considering fibrosis can protect the ischemic heart from ventricular wall rupture in the acute phase of MI, miR-409-3p inhibition treatment should be carefully assessed in the early stage of MI [34]. A comprehensive study that tracked miR-409-3p circulation levels in patients with cardiac fibrosis for a long time was also recommended to investigate the potential diagnostic roles of miR-409-3p.

The proliferation of fibroblast and fibroblast-to-myofibroblast transition are two significant events of cardiac fibrosis [10]. Our experiments in vitro proved that miR-409-3p could promote fibroblast proliferation and FMT characterized by the presence of *α*-SMA, a hallmark of the cardiac fibrotic response [35]. Overexpression of miR-409-3p enhanced the fibrosis degree of NRCF at the basal level, while upregulation of miR-409-3p failed to further advance fibrosis levels after treatment with Ang II or TGF-*β*, probably due to the fact that Ang II or TGF-*β* stimulation had brought NRCF to the highest degree of fibrosis. Inhibition of miR-409-3p decreased but did not abolish the cardiac fibrosis triggered by Ang II or TGF-*β*, indicating that other molecules are involved in this process. Since many miRNAs were irregular in our initial miRNA sequencing analysis, the interaction between these molecules constitutes a complex regulatory network, which can be explored later.

This present study also expounded on the cardiac fibrosis-related upstream and downstream genes of miR-409-3p. Neither of them has been discussed in cardiac fibrosis to date. We proposed a new candidate called Gpd1, which was reported to inhibit the proliferation of tumor cells [22, 23]. Gpd1 was demonstrated to be a direct target of miR-409-3p by luciferase assays and negatively regulated by miR-409-3p in this study. Results showed that reduction of Gpd1 via siRNA abolished the antifibrotic effect of the miR-409-3p inhibitor on NRCF, supporting our hypothesis that the profibrotic effect of miR-409-3p was at least partially attributed to Gpd1 suppression. However, a significant difference is still observed between the NRCFs transfected with siRNA Gpd1 alone and in the presence of siRNA Gpd1+miR-409-3p inhibitor. This result probably due to that miR-409-3p inhibitor led to higher level of Gpd1, attenuating the effect of siRNA Gpd1. So the siRNA Gpd1 is less efficient in NRCFs transfected with Gpd1+miR-409-3p inhibitor compared to the NRCFs transfected with siRNA Gpd1 alone.

Furthermore, we figured out GATA2 as a positive regulatory TF of miR-409-3p from the TransmiR database. GATA2 overexpression increased miR-409-3p levels in western blot assays but decreased GATA2 had the opposite effect. Zinc finger transcription factor GATA2 is crucial for the differentiation and proliferation of endothelial and immature hematopoietic cells [36]. It also protects against heart failure during pressure overload [37]. However, its role in cardiac fibrosis remains unknown. We further found that inhibition of miR-409-3p attenuated the profibrotic effect of GATA2 overexpression plasmid in NRCF, supporting our hypothesis that GATA2 is a potential positive fibrosis-associated regulatory TF of miR-409-3p. More rigorous functional studies about the relationships between miR-409-3p and Gpd1 or GATA2 in vivo and in vitro are critical to be perfected in the future to search for precise therapeutic targets.

## 5. Conclusions

In conclusion, the current study identifies a novel biological function of miR-409-3p that promotes cardiac fibrosis by directly targeting the downstream gene Gpd1. Additionally, GATA2 is a fibrosis-associated positive regulatory transcription factor of miR-409-3p. These findings propose a potential candidate for therapeutic intervention in cardiac fibrosis.

## Data Availability

The data used to support the findings of this study are available from the corresponding author upon request.

## Supplementary Materials

Supplementary Materials Table 1: sequences of primers. The primers used for qPCR are listed in this table; 18S was used as an endogenous control. Table 2: primary and secondary antibodies for western blot analysis. Details of the all antibodies used for western blot are listed in this table. Table 3: dysregulated microRNAs between ventricular tissues of MI mice and sham mice. MicroRNA sequencing analysis showed that 104 miRNAs were upregulated and 34 were downregulated; all these changes were more than twice, and *P* value was less than 0.05. Table 4: 36 potential target genes of miR-409-3p predicted by 3 miRNA target prediction websites. Intersection of three databases (miRDB, TargetScan, and miRWalk) used for predicting potential targets of miR-409-3p. Table 5: dysregulated mRNAs between ventricular tissues of MI mice and sham mice. 16 genes of dysregulated mRNAs from mRNA sequencing analysis, 8 genes were upregulated and 8 were downregulated; all these changes were more than twice, and *P* value was less than 0.05.
