# Supplementary material for: miR-409-3p Regulated by GATA2 Promotes Cardiac Fibrosis through Targeting Gpd1
Source: Oxid Med Cell Longev. 2022 Oct 12;2022:8922246. doi: 10.1155/2022/8922246 (PMC9581711; doi:10.1155/2022/8922246)
Supplement: Supplementary Materials — Table 1: sequences of primers. The primers used for qPCR are listed in this table; 18S was used as an endogenous control. Table 2: primary and secondary antibodies for western blot analysis. Details of the all antibodies used for western blot are listed in this table. Table 3: dysregulated microRNAs between ventricular tissues of MI mice and sham mice. MicroRNA sequencing analysis showed that 104 miRNAs were upregulated and 34 were downregulated; all these changes were more than twice, and P value was less than 0.05. Table 4: 36 potential target genes of miR-409-3p predicted by 3 miRNA target prediction websites. Intersection of three databases (miRDB, TargetScan, and miRWalk) used for predicting potential targets of miR-409-3p. Table 5: dysregulated mRNAs between ventricular tissues of MI mice and sham mice. 16 genes of dysregulated mRNAs from mRNA sequencing analysis, 8 genes were upregulated and 8 were downregulated; all these changes were more than twice, and P value was less than 0.05. [file 8922246.f1.docx]

**Supplementary Materials**

**Table 1: Sequences of primers**

| **Gene** | **Species** | **Forward** | **Reverse** |
| --- | --- | --- | --- |
| 18s | mouse | TCAAGAACGAAAGTCGGAGG | GGACATCTAAGGGCATCAC |
| α-SMA | mouse | GTCCCAGACATCAGGGAGTAA | TCGGATACTTCAGCGTCAGGA |
| Col1a1 | mouse | TCTAGACATGTTCAGCTTTGTGGAC | TCTGTACGCAGGTGATTGGTG |
| Col3a1 | mouse | CTGTAACATGGAAACTGGGGAAA | CCATAGCTGAACTGAAAACCACC |
| 18s | rat | TCAAGAACGAAAGTCGGAGG | GGACATCTAAGGGCATCAC |
| α-SMA | rat | GTCCCAGACATCAGGGAGTAA | TCGGATACTTCAGCGTCAGGA |
| Col1a1 | rat | GAGCGGAGAGTACTGGATCGA | CTGACCTGTCTCCATGTTGCA |
| Col3a1 | rat | TGCCATTGCTGGAGTTGGA | GAAGACATGATCTCCTCAGTGTTGA |
| Gpd1 | rat | ATGGCTGGCAAGAAAGTCTG | CCTGCATTGCTACCCACGAT |

**Table 2: Primary and secondary antibodies for Western blot analysis**

| Antibody | Dilution | Amount of proteins | % of  acrylamide gel | Blocking solution | Provider |
| --- | --- | --- | --- | --- | --- |
| α-SMA | 1:1000 | 20 μg | 10 | 5% milk | Abclonal (Wuhan, China) |
| Col1a1 | 1:1000 | 20 μg | 10 | 5% milk |  |
| Col3a1 | 1:1000 | 20 μg | 10 | 5% milk |  |
| CTGF | 1:500 | 20 μg | 10 | 5% milk |  |
| Gpd1 | 1:500 | 20 μg | 10 | 5% milk |  |
| GATA2 | 1:500 | 20 μg | 10 | 5% milk |  |
| GAPDH | 1:2000 | 20 μg | 10 | 5% milk |  |
| β-actin | 1:2000 | 20 μg | 10 | 5% milk |  |
| HRP-linked goat anti-rabbit IgG | 1:5000 | / | / | / |  |

**Table 3:** **Dysregulated microRNAs between ventricular tissues of MI mice and sham mice**

| **miRNA** | **Regulation** | **Fold change** | **P value** |
| --- | --- | --- | --- |
| **miR-34c-5p** | ↑ | 19.17705 | 8.22E-24 |
| miR-34b-3p | ↑ | 15.43478 | 1.25E-08 |
| miR-214-3p | ↑ | 12.26971 | 3.30E-16 |
| **miR-214-5p** | ↑ | 11.90878 | 1.15E-19 |
| miR-21a-5p | ↑ | 11.14546 | 7.20E-11 |
| miR-299a-5p | ↑ | 11.02857 | 1.70E-07 |
| **miR-329-3p** | ↑ | 10.38235 | 4.09E-08 |
| miR-34c-3p | ↑ | 10.2 | 2.19E-06 |
| miR-337-5p | ↑ | 9.607595 | 3.51E-08 |
| miR-409-5p | ↑ | 9.484848 | 1.39E-06 |
| miR-147-3p | ↑ | 9.285714 | 2.53E-05 |
| miR-140-3p | ↑ | 8.98371 | 1.98E-15 |
| miR-206-3p | ↑ | 8.727273 | 0.003594 |
| miR-434-5p | ↑ | 8.466527 | 1.50E-15 |
| miR-199a-5p | ↑ | 8.454161 | 9.13E-11 |
| miR-199a-3p | ↑ | 8.2389 | 1.75E-11 |
| miR-379-5p | ↑ | 8.163498 | 1.75E-13 |
| **miR-199b-3p** | ↑ | 8.149942 | 1.99E-11 |
| **miR-455-5p** | ↑ | 7.97561 | 6.18E-10 |
| miR-495-3p | ↑ | 7.87931 | 9.06E-07 |
| miR-382-5p | ↑ | 7.849057 | 1.82E-06 |
| miR-21c | ↑ | 7.669355 | 1.56E-08 |
| miR-31-3p | ↑ | 7.454545 | 0.001455 |
| miR-434-3p | ↑ | 7.238754 | 1.10E-09 |
| **miR-134-5p** | ↑ | 6.858025 | 8.41E-08 |
| **miR-224-5p** | ↑ | 6.857143 | 0.000251 |
| miR-541-5p | ↑ | 6.784483 | 1.55E-11 |
| miR-455-3p | ↑ | 6.619048 | 2.69E-06 |
| **miR-410-3p** | ↑ | 6.56 | 4.68E-05 |
| miR-376a-5p | ↑ | 6.5 | 0.003103 |
| **miR-431-5p** | ↑ | 6.4 | 4.91E-06 |
| miR-380-5p | ↑ | 6.321429 | 0.001565 |
| miR-125b-1-3p | ↑ | 6.288 | 1.53E-08 |
| miR-329-5p | ↑ | 6.269231 | 0.000175 |
| miR-136-3p | ↑ | 6.243243 | 0.00051 |
| **miR-574-3p** | ↑ | 6.088825 | 1.75E-08 |
| miR-199b-5p | ↑ | 6.064516 | 1.64E-08 |
| miR-140-5p | ↑ | 5.996899 | 2.33E-08 |
| miR-127-5p | ↑ | 5.857143 | 0.008771 |
| **miR-411-5p** | ↑ | 5.853289 | 9.49E-10 |
| miR-370-3p | ↑ | 5.813953 | 1.78E-05 |
| miR-147-5p | ↑ | 5.636364 | 0.0156 |
| miR-298-5p | ↑ | 5.571429 | 0.00411 |
| miR-183-5p | ↑ | 5.56 | 0.000457 |
| miR-127-3p | ↑ | 5.356362 | 1.75E-10 |
| **miR-379-3p** | ↑ | 5.3125 | 0.000195 |
| miR-3081-3p | ↑ | 5.171875 | 0.000103 |
| miR-6944-3p | ↑ | 5.1 | 0.001611 |
| **miR-381-3p** | ↑ | 5.049016 | 4.35E-08 |
| miR-369-5p | ↑ | 4.880597 | 6.93E-05 |
| miR-146b-5p | ↑ | 4.867834 | 6.84E-08 |
| miR-668-3p | ↑ | 4.866667 | 0.005415 |
| miR-299a-3p | ↑ | 4.828125 | 0.000224 |
| miR-154-5p | ↑ | 4.764706 | 0.00379 |
| **miR-31-5p** | ↑ | 4.631579 | 0.000475 |
| miR-344d-3p | ↑ | 4.428571 | 0.041078 |
| miR-380-3p | ↑ | 4.4 | 0.006513 |
| **miR-409-3p** | ↑ | 4.226667 | 0.000135 |
| miR-182-5p | ↑ | 4.205128 | 0.000342 |
| **miR-673-5p** | ↑ | 4.2 | 0.008697 |
| miR-136-5p | ↑ | 4.19469 | 0.000106 |
| miR-369-3p | ↑ | 4.16 | 0.002099 |
| miR-411-3p | ↑ | 4.037037 | 0.000159 |
| miR-222-3p | ↑ | 4.008403 | 0.000286 |
| miR-376b-5p | ↑ | 3.864407 | 0.000424 |
| miR-433-3p | ↑ | 3.833333 | 0.012085 |
| miR-1a-1-5p | ↑ | 3.8 | 0.032149 |
| miR-96-5p | ↑ | 3.705882 | 0.001417 |
| miR-300-3p | ↑ | 3.60515 | 0.000523 |
| miR-341-3p | ↑ | 3.409091 | 0.030766 |
| miR-429-3p | ↑ | 3.344828 | 0.012558 |
| miR-543-3p | ↑ | 3.257426 | 0.004171 |
| miR-142a-5p | ↑ | 3.243094 | 0.03288 |
| miR-23b-3p | ↑ | 3.157512 | 0.000275 |
| miR-296-5p | ↑ | 3.142857 | 0.019339 |
| miR-501-3p | ↑ | 3.106195 | 0.002515 |
| miR-574-5p | ↑ | 3.089172 | 0.002841 |
| miR-218-5p | ↑ | 2.985651 | 0.002643 |
| miR-1839-5p | ↑ | 2.907692 | 0.01195 |
| miR-323-3p | ↑ | 2.9 | 0.007003 |
| miR-152-3p | ↑ | 2.882915 | 0.003679 |
| miR-21a-3p | ↑ | 2.8 | 0.007553 |
| miR-666-5p | ↑ | 2.765957 | 0.004315 |
| miR-142a-3p | ↑ | 2.765562 | 0.009207 |
| miR-320-3p | ↑ | 2.74505 | 0.000839 |
| miR-99b-3p | ↑ | 2.730375 | 0.022203 |
| miR-497a-5p | ↑ | 2.707207 | 0.024916 |
| miR-221-3p | ↑ | 2.587393 | 0.009171 |
| miR-299b-3p | ↑ | 2.583333 | 0.033678 |
| miR-132-3p | ↑ | 2.552083 | 0.038008 |
| miR-210-5p | ↑ | 2.4 | 0.024978 |
| miR-217-5p | ↑ | 2.4 | 0.007669 |
| miR-23a-3p | ↑ | 2.336085 | 0.024328 |
| miR-125b-5p | ↑ | 2.317388 | 0.029627 |
| miR-6945-3p | ↑ | 2.3 | 0.012672 |
| miR-146a-5p | ↑ | 2.286662 | 0.015039 |
| miR-223-3p | ↑ | 2.247059 | 0.045101 |
| miR-195a-5p | ↑ | 2.230835 | 0.020809 |
| miR-184-3p | ↑ | 2.218905 | 0.010599 |
| miR-15b-5p | ↑ | 2.109131 | 0.04499 |
| miR-103-3p | ↑ | 2.106527 | 0.028256 |
| miR-1193-3p | ↑ | 2.1 | 0.035508 |
| miR-146b-3p | ↑ | 2 | 0.033753 |
| miR-485-3p | ↑ | 2 | 0.033753 |
| miR-194-1-3p | ↓ | 2 | 0.020483 |
| miR-6516-3p | ↓ | 2 | 0.020102 |
| miR-1a-3p | ↓ | 2.012056 | 0.010983 |
| miR-30c-5p | ↓ | 2.069108 | 0.002443 |
| miR-133b-3p | ↓ | 2.096055 | 8.40E-05 |
| miR-201-3p | ↓ | 2.1 | 0.020635 |
| miR-30c-1-3p | ↓ | 2.1 | 0.004064 |
| miR-139-5p | ↓ | 2.137591 | 0.000187 |
| miR-9-5p | ↓ | 2.141213 | 0.001569 |
| miR-133c | ↓ | 2.173913 | 0.037279 |
| miR-378c | ↓ | 2.207969 | 2.92E-05 |
| miR-30c-2-3p | ↓ | 2.212541 | 0.002818 |
| miR-338-3p | ↓ | 2.266667 | 0.031558 |
| miR-504-5p | ↓ | 2.276295 | 0.001236 |
| miR-1934-5p | ↓ | 2.3 | 0.038404 |
| miR-378a-5p | ↓ | 2.374259 | 1.97E-05 |
| miR-6911-3p | ↓ | 2.375 | 0.022734 |
| miR-149-5p | ↓ | 2.393664 | 0.000118 |
| miR-204-5p | ↓ | 2.453586 | 6.86E-05 |
| miR-1843a-3p | ↓ | 2.506024 | 0.000319 |
| miR-30e-3p | ↓ | 2.511415 | 7.90E-05 |
| miR-9-3p | ↓ | 2.55 | 0.010889 |
| miR-1198-3p | ↓ | 2.583333 | 0.018675 |
| miR-208a-5p | ↓ | 2.585366 | 0.005274 |
| **miR-208a-3p** | ↓ | 2.60222 | 3.60E-07 |
| **miR-499-5p** | ↓ | 2.689264 | 1.73E-06 |
| miR-133a-5p | ↓ | 2.804878 | 0.002604 |
| miR-139-3p | ↓ | 2.897436 | 0.005581 |
| miR-181a-2-3p | ↓ | 2.967626 | 6.19E-05 |
| miR-6929-3p | ↓ | 3.1 | 0.002836 |
| miR-135a-5p | ↓ | 3.272727 | 2.74E-05 |
| **miR-378b** | ↓ | 3.381974 | 1.91E-05 |
| miR-881-3p | ↓ | 3.652174 | 4.11E-05 |
| miR-486a-3p | ↓ | 3.666667 | 0.000501 |

**Table 4: 36 potential target genes of miR-409-3p predicted by 3 miRNA target prediction websites**

| **Number** | **Gene Symbol** | **Gene Description** |
| --- | --- | --- |
| 1 | Mtss1 | I-BAR domain containing |
| 2 | St18 | C2H2C-type zinc finger |
| 3 | Mme | membrane metallo-endopeptidase |
| 4 | Rorb | RAR-related orphan receptor B |
| 5 | Tpsg1 | tryptase gamma 1 |
| 6 | Pkd2l2 | polycystin 2 like 2 |
| 7 | Psmc6 | proteasome 26S subunit, ATPase 6 |
| 8 | Ms4a2 | membrane spanning 4-domains A2 |
| 9 | Cst9l | cystatin 9-like |
| 10 | Xirp2 | xin actin-binding repeat containing 2 |
| 11 | Cntn4 | contactin 4 |
| 12 | Arl5b | ADP-ribosylation factor like GTPase 5B |
| 13 | Pfkfb4 | 6-phosphofructo-2-kinase/fructose-2,6-biphosphatase 4 |
| 14 | Lamp1 | lysosomal-associated membrane protein 1 |
| 15 | Dixdc1 | DIX domain containing 1 |
| 16 | Ss18 | nBAF chromatin remodeling complex subunit |
| 17 | Hsf4 | heat shock transcription factor 4 |
| 18 | Pcsk7 | proprotein convertase subtilisin/kexin type 7 |
| 19 | Sh3bgrl3 | SH3 domain binding glutamate-rich protein like 3 |
| 20 | Zeb1 | zinc finger E-box binding homeobox 1 |
| 21 | Ube4b | ubiquitination factor E4B |
| 22 | Matr3 | matrin 3 |
| 23 | Stk32c | serine/threonine kinase 32C |
| 24 | Usp9x | ubiquitin specific peptidase 9, X-linked |
| 25 | Igsf11 | immunoglobulin superfamily, member 11 |
| 26 | Icos | inducible T-cell co-stimulator |
| 27 | Socs6 | inducible T-cell co-stimulator |
| 28 | **Gpd1** | glycerol-3-phosphate dehydrogenase 1 |
| 29 | Gnrhr | gonadotropin releasing hormone receptor |
| 30 | Rb1cc1 | RB1-inducible coiled-coil 1 |
| 31 | Rps6kb1 | ribosomal protein S6 kinase B1 |
| 32 | Rtn3 | reticulon 3 |
| 33 | Tnp2 | transition protein 2 |
| 34 | Txlnb | taxilin beta |
| 35 | Lum | lumican |
| 36 | Btbd3 | BTB domain containing 3 |

**Table 5:** **Dysregulated mRNAs between ventricular tissues of MI mice and sham mice**

| **mRNA** | **Gene Description** | **Regulation** | **Fold change** | **P value** |
| --- | --- | --- | --- | --- |
| Col8a2 | collagen type VIII alpha 2 | ↑ | 124.6209 | 5.39E-48 |
| Mmp12 | matrix metallopeptidase 12 | ↑ | 65.93196 | 2.5E-05 |
| Ak5 | adenylate kinase 5 | ↑ | 38.28465 | 1.05E-08 |
| Slc41a2 | solute carrier family 41 member 2 | ↑ | 23.9903 | 0.001475 |
| Ctgf | connective tissue growth factor | ↑ | 11.60055 | 5.2E-27 |
| Nlrp1b | NLR family pyrin domain containing 1B | ↑ | 6.115172 | 2.36E-05 |
| Atp8b1 | ATPase class I type 8B member 1 | ↑ | 3.469109 | 6.85E-07 |
| Psip1 | PC4 and SFRS1 interacting protein 1 | ↑ | 2.579578 | 4.23E-06 |
| Slc4a3 | solute carrier family 4 (anion exchanger) member 3 | ↓ | 2.466559 | 3.51E-05 |
| Mrpl32 | mitochondrial ribosomal protein L32 | ↓ | 2.575085 | 9.31E-12 |
| Gys1 | glycogen synthase 1 | ↓ | 2.719667 | 0.000152 |
| Gcdh | glutaryl-Coenzyme A dehydrogenase | ↓ | 2.753526 | 2.67E-06 |
| Tbx5 | T-box 5 | ↓ | 2.761649 | 3.16E-09 |
| Fgf13 | fibroblast growth factor 13 | ↓ | 2.812455 | 3.93E-06 |
| **Gpd1** | glycerol-3-phosphate dehydrogenase 1 | ↓ | 2.855414 | 4.41E-05 |
| Sfrp5 | secreted frizzled-related sequence protein 5 | ↓ | 16.96348 | 1.65E-05 |
